# Dental Chemistry of the Mouth

**Published:** 1857-09

**Authors:** Geo. Watt


### DENTAL CHEMISTRY OF THE MOUTH.

BY GEO. WATT.

At the close of our article on MUCUS, we proposed in our next to consider the various articles which, in ordinary life, exert an injurious chemical influence on the teeth. We did not then properly estimate the magnitude of our promise, nor appreciate the numberless difficulties that lay in our way. Having come to our senses in this respect, we now only propose to do something, if we can, toward the fulfillment of our promise.

To properly understand any chemical action to which the teeth are subject, it is necessary to bear in mind their texture and composition, and to consider the chemical properties of at least their principal constituents. It must also be remembered that the teeth are endowed with vitality. As dental caries, the most common disease of the human race, is now universally conceded to be the result of chemical action, the importance of this subject is at once manifest. The time is not far distant when, in every case of recent caries, the enlightened practitioner will be able, by the character of the decay and the habits and constitution of the patient, to detect and identify the agent or agents producing the disease. Any practice short of this knowledge must be, at least to some extent, guesswork, and is, although the best we can now do, empirical practice.

The fact that an active alkaline base is the principal inorganic ingredient of the teeth would indicate clearly that their great danger lies in the presence of acids; and all experience demonstrates the truth of this inference. This danger is also greater from the fact that the principal salt of this base, present in the tooth substance, combines with several acids without undergoing decomposition.

It is evident that the acids do not all act alike on the teeth. Indeed, some exert no influence whatever on them, while others act with great energy on each and all of their constituents. It would be an endless task to consider all the subtances which are capable of exerting an injurious chemical influence on the teeth; and, perhaps, it would be as unprofitable as endless. All that is now aimed at is an accurate account of the various substances which ordinarily act chemically on the teeth—which produce caries and "chemical abrasion."

But without farther preface, we will proceed to notice some of the chemical agents alluded to.

NITRIC ACID.—This acid is composed of five equivalents of oxygen united with one of nitrogen. Its symbol is, therefore, $N\,O._5$ It acts with great energy on all the constituents of the tooth. Its great energy of action depends on a variety of circumstances. As an acid, it unites energetically with bases, and will, therefore, take the lime and kindred bases from the weaker acids. From its ready decomposition, it affords oxygen, in its nascent condition, for the destruction of oxydizable substances. Its action on the tooth may be thus briefly described: It dissolves the phosphate of lime, decomposes the carbonate, setting the carbonic acid free, and forming nitrate of lime, and destroys the organic portion, producing a highly softened state of the carious matter. In fact, it is a prominent, if not the principal agent in the production of the "white decay."

But the question naturally arises, Is an agent so destructive in its tendencies likely to come in contact with the teeth, and if so, under what circumstances? The question is important, and the answer, perhaps, difficult.

It is well known that this acid is frequently administered as a tonic; and it is a lamentable fact that far too little attention is paid to the prevention of its injurious effects on the teeth in such cases, but this will by no means account for the frequency with which it evidently injures the dental organs. A few thoughts in regard to its formation may throw some light on the subject.

It is a singular fact that though nitrogen and oxygen manifest but little affinity for each other, yet they unite in various proportions, forming at least five well known distinct compounds. It appears, however, from a variety of circumstances, that their tendency is to unite in the proportions which form nitric acid. The protoxyd is readily decomposed, and yields nitrogen, oxygen, and *nitrous acid.* The binoxyd, if brought in contact with the atmosphere, takes from it two equivalents of oxygen, and also becomes *nitrous acid*, or $N\,O_4$. Hyponitrous acid, $N\,O_3$, on admixture with water, is converted into nitric acid and binoxyd of nitrogen, thus: $3N\,O_3 = N\,O_5 + 2N\,O_2$, in which case the latter will be converted into nitrous acid, which, in the presence of water, is converted into *nitric acid* and binoxyd of nitrogen.

It follows from this that, if oxygen and nitrogen unite at all in the mouth, let the proportions be, at the first, what they will, nitric acid must be the ultimate result—as air and moisture, the only agents necessary in the transformation, are here always present.

The reader will now think of the mucus, and particles of nitrogenous food lodged about the teeth undergoing decomposition, and yielding nitrogen to the oxygen of the atmosphere, or of the fluids of the mouth, and will conclude that all is explained. Well, perhaps it is. But let us consider. Nitrogen is emphatically a "conservative" element, and manifests but little tendency to unite with anything, and especially with oxygen. It is probable, therefore, that these two elements unite indirectly. It should be borne in mind that organic nitrogenous bodies contain hydrogen and oxygen, as well as nitrogen. Consequently, by their decomposition, these elements are all liberated. The mutual affinities of hydrogen and nitrogen take precedence, and the result is the formation of ammonia, $N\,H_3$. But ammonia, exposed to the action of oxygen, is always decomposed; an oxyd of nitrogen being formed, and of course *nitric acid* is the result.

With this view of the case, and from the fact that many persons permit the buccal mucus, as well as particles of nitrogenous food, to remain around, upon, and between the teeth, till decomposition is effected, it is not surprising that the white variety of dental caries is so frequently found.

Nitric acid is also sometimes formed in the mouth by the agency of galvanic action. When two metals are placed in the mouth in proximity to each other, and the fluids of the mouth are capable of acting on one of them, galvanic action is established. And if they are so situated that the mucous membrane forms a connecting conductor, by being in contact with both, especially if the metallic surfaces be considerable, a current is established, sufficient to decompose any of the binary compounds contained in these fluids. The liberated nitrogen, hydrogen, and oxygen will result, as above, in the formation of ammonia, and then, nitric acid. But galvanic action in the mouth is more likely to develop hydrochloric, than nitric acid. This will be noticed again.

SULPHURIC ACID.—Sulphuric acid is composed of 16 parts of sulphur united with 24 of oxygen. Its symbol is, therefore, $S\,O_3$. In addition to those properties which characterize it as an acid, it is a powerful caustic poison, and promptly destroys the various tissues with which it comes in contact. Its chemical action on ordinary tissues depends principally on its affinity for water, but not altogether; for it has the ability to coagulate and unite with albumen, and to dissolve fibrin. In common with other acids, it has a strong affinity for alkaline bases.

With these properties in view, let us examine its action on the teeth.

The affinity of this acid for water is so energetic that it seems even to force its elements to forsake favorite combinations, and to unite with each other, that it may be gratified. For example, a cork in a bottle of sulphuric acid becomes dark colored, and is really charred. Now, a cork, like other wood, is mainly composed of carbon, hydrogen, and oxygen—the two latter being in the proper proportions to form water. Their affinity for each other, quickened by that of the acid for the result of their combination, causes them to forsake the carbon, unite with each other to form water, and then combine with the acid. The same phenomena occur when it acts on animal tissues; for they are principally composed of the above named elements, with the addition of nitrogen. Accordingly, "black spots are frequently observed in the stomachs of those who have swallowed the acid." Now, that its slow and prolonged action on the gelatinous portion of the tooth would result in its carbonization is a conclusion justified both by inference and experiment. But carbonized gelatin is "animal charcoal," the color of which is a prominent characteristic of "black decay."

The phosphate of lime in the tooth, which is not the neutral, but a subphosphate, is not soluble in sulphuric acid; nor is the acid capable of decomposing it, except in the presence of alcohol. It follows, then, that this acid does not break down the texture of the tooth to the extent that some others do, simply because it cannot unite with, or, under ordinary circumstances, decompose the principal earthy salt of which it is composed. And here we have a second characteristic of "black decay."

It is now time to inquire whether at all, and if so, by what means, and under what circumstances this acid is brought in contact with the dental organs.

Sulphuric, like nitric acid is frequently administered as a medicine, and generally with criminal negligence in regard to its action on the teeth. But we cannot regard this as the only or principal source of danger from this acid. If oxygen unites at all with sulphur, the tendency, under ordinary circumstances, is to the formation of sulphuric acid, as sulphurous acid, in the presence of moisture, is rapidly converted into the sulphuric. The whole question, then, is reduced to this,—Is sulphur ordinarily present in the mouth, and liable there to become oxydized?

Albumen is a constituent of mucus, and is contained in many articles of food. Sulphur, if not a constituent of, is always united with albumen. Its ordinary presence in the mouth is, therefore, easily explained. Sulphur and oxygen unite directly, under various circumstances, as in the combustion of sulphur; but it is probable that the union here is effected by indirect means. Hydrosulphuric acid, or sulphureted hydrogen, is one of the results of the putrefactive decomposition of albuminous substances. The breaths of our patients often bear ample testimony to its presence in the mouth. Now, the oxygen of the atmosphere rapidly decomposes this acid, by taking its hydrogen to form water. The sulphur is, therefore, set free, and being in the nascent state, its affinities are increased in energy, and it also unites with oxygen, forming sulphurous acid, $S\,O_2$, which in the presence of the water of the saliva, is rapidly converted into sulphuric acid, or $S\,O_3$.

The quantity of sulphur present in the mouth at any one time is very minute; and a great proportion of this is exhaled by the breath, before it has time to undergo decomposition. And sulphuric acid, as already noticed, has a weaker affinity for the constituents of the tooth than some others. Hence, "black decay" is not so frequently met with as some other varieties. And as, from the nature of the chemical action, the texture of the tooth is not so entirely broken up, the carbonized portion protects the parts beneath it. This variety of decay, therefore, progresses less rapidly than others.

HYDROCHLORIC ACID.—This acid is also called chlorohydric, and muriatic acid. It is composed of 35 parts of chlorine, united with 1 of hydrogen. Its symbol is HCl. Though its elements manifest a strong affinity for each other, yet it is very readily decomposed; and many of its chemical manifestations result from the action of one or both of its liberated elements. It is on this principle the acid attacks metals—being decomposed the chlorine unites with the metal to form a chloride, and the hydrogen escapes with effervescence.

This acid, like those previously considered, is a caustic poison. Its escharotic power depends, mainly, on its affinity for water, which is very active; and on its ability to coagulate albumen. Its chemical action is generally inferior to that of the two acids just considered. It unites with bases, forming a class of salts called hydrochlorates; and sometimes it combines with a salt without decomposing it, or being itself decomposed. When concentrated, it dissolves animal tissues, but is, in this respect, far inferior to nitric acid. When much diluted and mixed with dried mucous membrane, it dissolves coagulated albumen, fibrin, &c., performing, to all appearance, an artificial digestion.

A careful observation of these properties will enable us to understand the action of this acid on the tooth.

The carbonate of lime and the acid are mutually decomposed. The results are chloride of calcium, water, and carbonic acid. The decomposition may be represented by the following equation:—

$$Ca\,O,\,C\,O_2,+HCl=Ca\,Cl+H\,O+C\,O_2.$$

The carbonic acid, of course, escapes as a gas, and the chloride, being very soluble, is dissolved in the saliva, and thus removed from the tooth.

The phosphate of lime, (bone phosphate) though not decomposed by, is highly soluble in hydrochloric acid. It is dissolved, and is thus removed from the organic portion of the tooth.

We have seen that this acid, unless highly concentrated, is not capable of dissolving the animal portion of the tooth. As this concentration is not likely to take place in the mouth, it follows that, when hydrochloric acid is the cause of dental caries, the earthy portion is dissolved and removed, while the animal portion principally remains in the carious cavity. And here we have the prominent characteristics of a third variety of decay.

I have not taken into the account any of the earthy salts contained in the tooth, but the phosphate and carbonate of lime. They are present in such small quantities that they exert but little influence on any of the chemical actions which we have considered.

Hydrochloric acid is also administered as a medicine; and the remarks made on the preceding acids apply equally here. This acid is an ingredient of the gastric fluid, and is often present, in abnormal quantities, in the stomach, from which it is thrown into the mouth by eructation and vomiting. But we can not thus account satisfactorily for the frequency with which the dental organs are evidently injured by this acid.

Though in its normal state the saliva is alkaline, yet in a variety of abnormal conditions it contains one or more free acids; and the hydrochloric is one of those most frequently present. It often originates, no doubt, in the decomposition of the soluble chlorides contained in the saliva and mucus. When the chlorine of these is liberated, it takes hydrogen from the water of the saliva, and this acid is a result of the union.

But, sometimes, hydrochloric acid is directly furnished by the salivary glands, either as a secretion or an excretion. The system may contain just its normal quantity of chlorine, but, if there be a deficiency of sodium or potassium, the relative excess of chlorine is converted into hydrochloric acid. In this case the acid is secreted. Or the quantity of potassium and sodium may be normal, with an excess of chlorine. The excess will unite, as before, with hydrogen, and the acid will be excreted. At all events, this acid is usually found in the mouth when the mucous membrane is inflamed, as well as in patients who indulge in the excessive use of salted meats.

Galvanic currents in the mouth always result in the formation of this acid. The chlorides of sodium and potassium, present in normal mucus and saliva, are decomposed, and their chlorine unites with hydrogen derived from the water of the saliva. It is on this principle that we frequently find a decayed surface around a gold filling, which is in close proximity with one of a different metal, or with a silver plate or clasp. In such decays, the animal portion usually remains, while the earthy portion is removed, just as would be expected from the prolonged action of dilute hydrochloric acid.

In these observations we have endeavored to set forth the results of the ordinary uninterrupted action of these acids on the teeth; and we have seen that they are capable of producing the three varieties of decay usually described, though we by no means maintain that they are the only agents capable of causing these results. Their actions, and consequently the characteristics of decay produced by them, are doubtless much modified by circumstances. One of them may be the destructive agent in the commencement of the caries, and, in process of time, another may be developed, and exert its specific influence on the same cavity. Then the phenomena would, of course, be complex. Again, it should be remembered that a strong affinity for water is a property common to all of them. It is possible, therefore, that carbonization, or blackening, may result from the action of any of them, yet it is by no means probable, at least with nitric acid.

But this paper is long enough. We originally intended to include in it the action of several organic acids, but our knowledge of them was too limited; and our experiments with them are not sufficiently matured to enable us to state results with any degree of confidence. Indeed, we find it difficult to conduct some experiments properly, on account of the frequent interruptions incident to the practical duties of the profession. We hope our readers will be patient; but, if any one is not, we would be glad to have him turn his impatience to a good account, by anticipating us in our experiments, and giving his deductions to the profession.

